# Supplementary material for: A neural network-based method for exhaustive cell label assignment using single cell RNA-seq data
Source: Sci Rep. 2022 Jan 18;12:910. doi: 10.1038/s41598-021-04473-4 (PMC8766435; doi:10.1038/s41598-021-04473-4)
Supplement: Supplementary file 1 — Supplementary Figures. [file 41598_2021_4473_MOESM1_ESM.pdf]

A neural-network based method for exhaustive cell label assignment using single cell RNA-seq data
Supplementary Material

Ziyi Li, Hao Feng

Numerical experiments with cell-sorted PBMC data

Supplementary Figure 1 and 2 show NeuCA's performance under various proportions of training and testing scenarios.

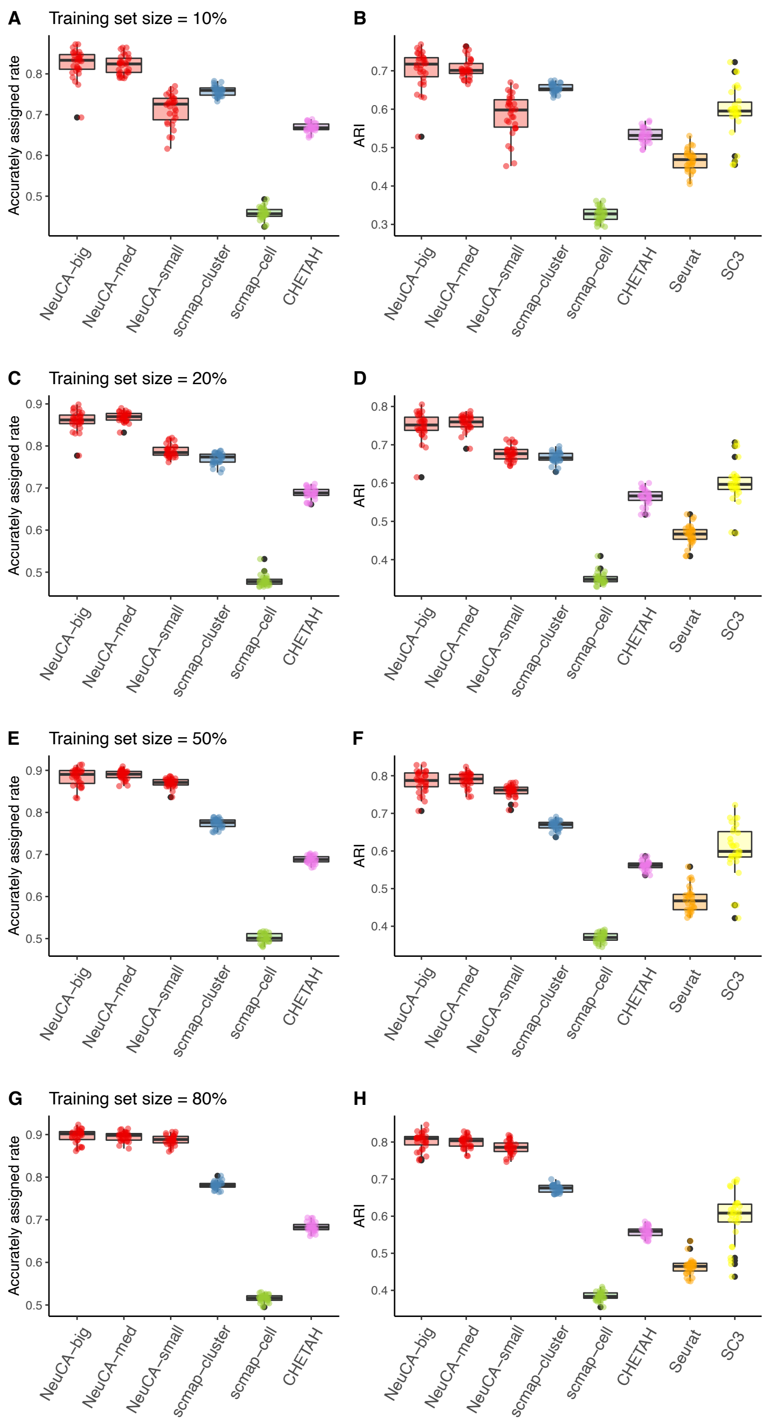

Supplementary Figure 1: Accurately assigned rate and ARI value using PBMC data with 8 cell types and different training set sizes. From top to bottom, the training set size increases from 10%, 20%, 50%, to 80%. Results are summarized over different testing size with each setting consisting of 20 Monte Carlo simulations.

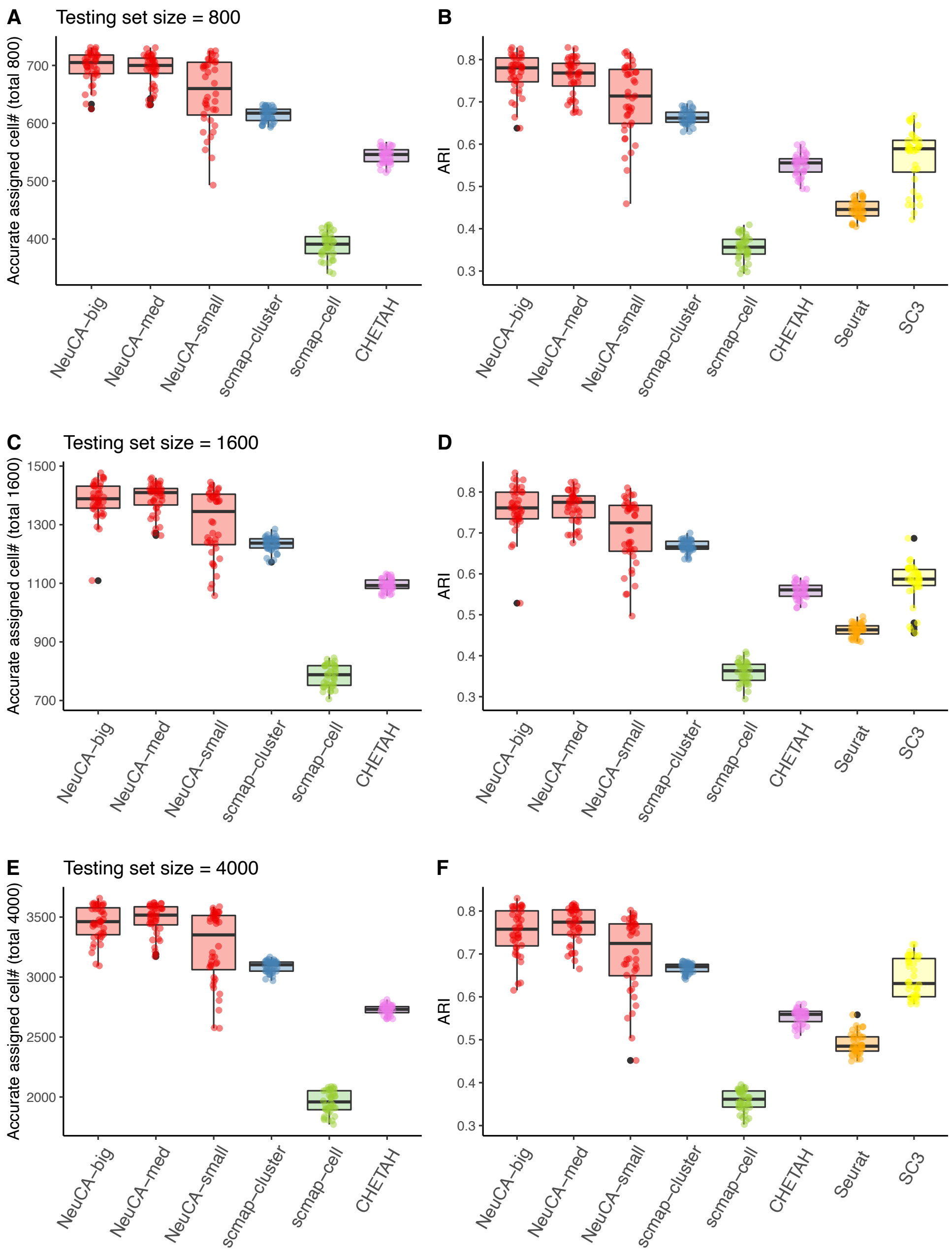

Supplementary Figure 2: Accurately assigned rate and ARI value using PBMC data with 8 cell types and different testing set size. From top to bottom, the testing set size increases from 800, 1600, to 4000. Results are summarized over different training size with each setting consisting of 20 Monte Carlo simulations.

Numerical experiments exclusively for T cells

Supplementary Figure 3 to 5 show NeuCA's performance at various split sizes of training and testing datasets, within hard-to-distinguish T cell subtypes.

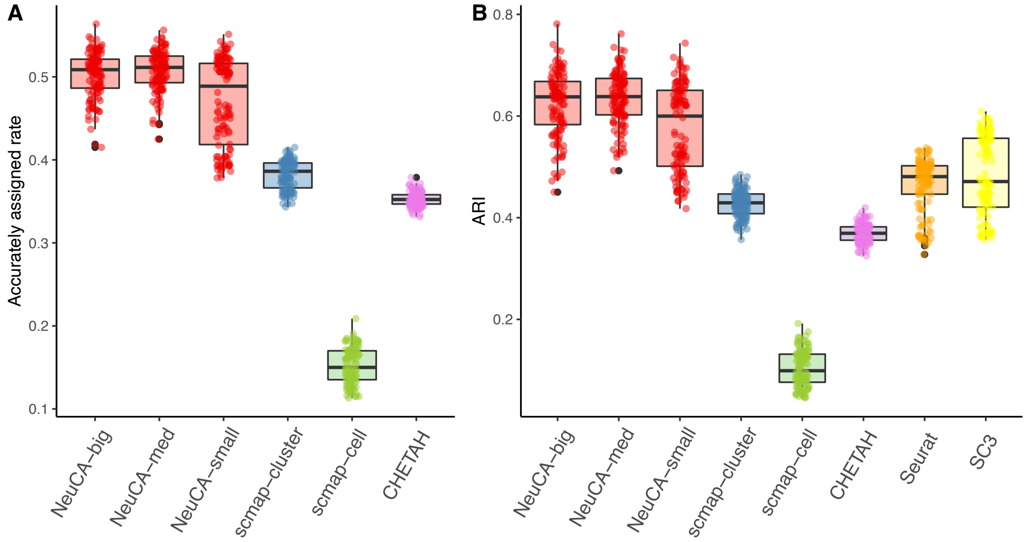

Supplementary Figure 3: Accurately assigned rate and ARI for applying the proposed method and existing methods on T-cell only dataset. Different training set sizes and testing set sizes are considered and summarized into one box for each method. 20 Monte Carlo simulations are used for each training/testing setting.

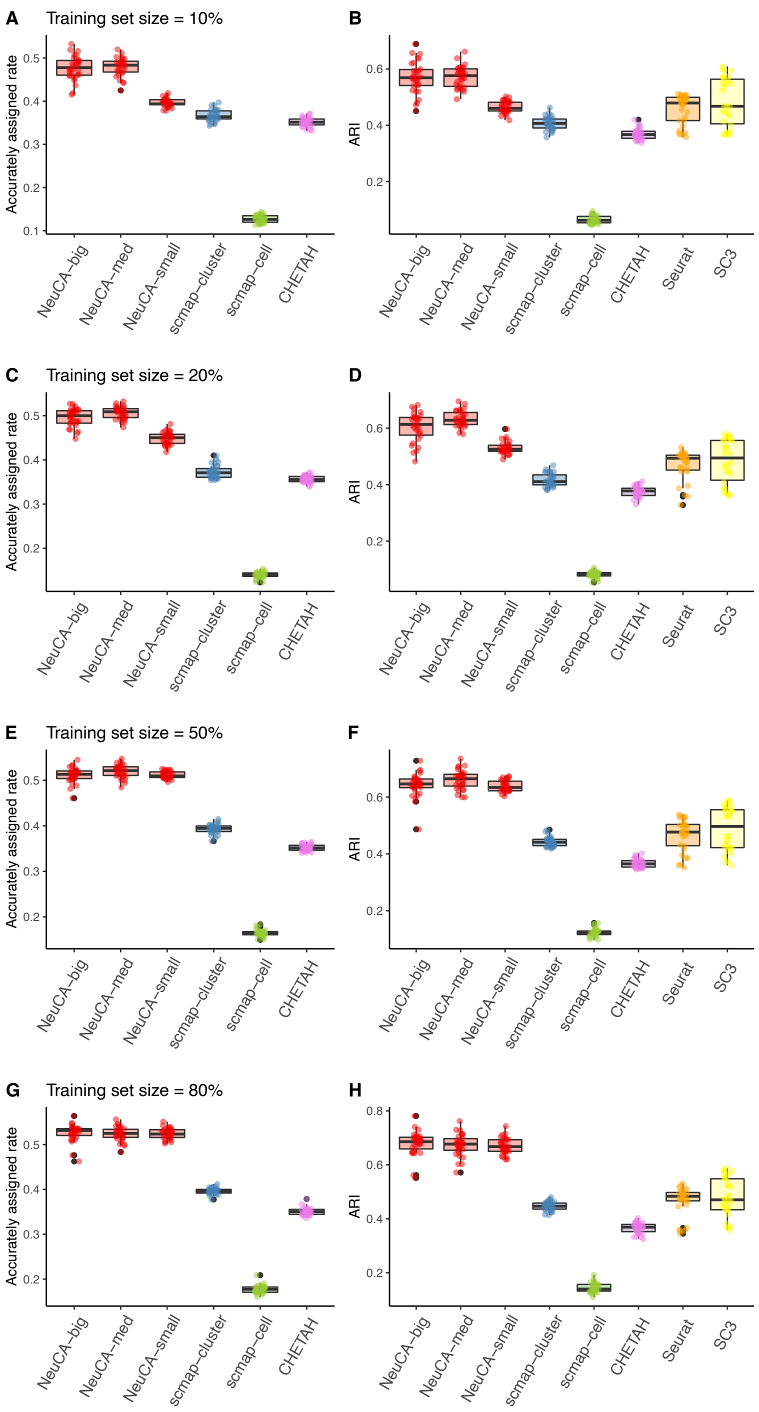

Supplementary Figure 4: Accurately assigned rate and ARI value using T-cell only data and different training set sizes. From top to bottom, the training set size increases from 10%, 20%, 50%, to 80%. Results are summarized over different testing size with each setting consisting of 20 Monte Carlo simulations.

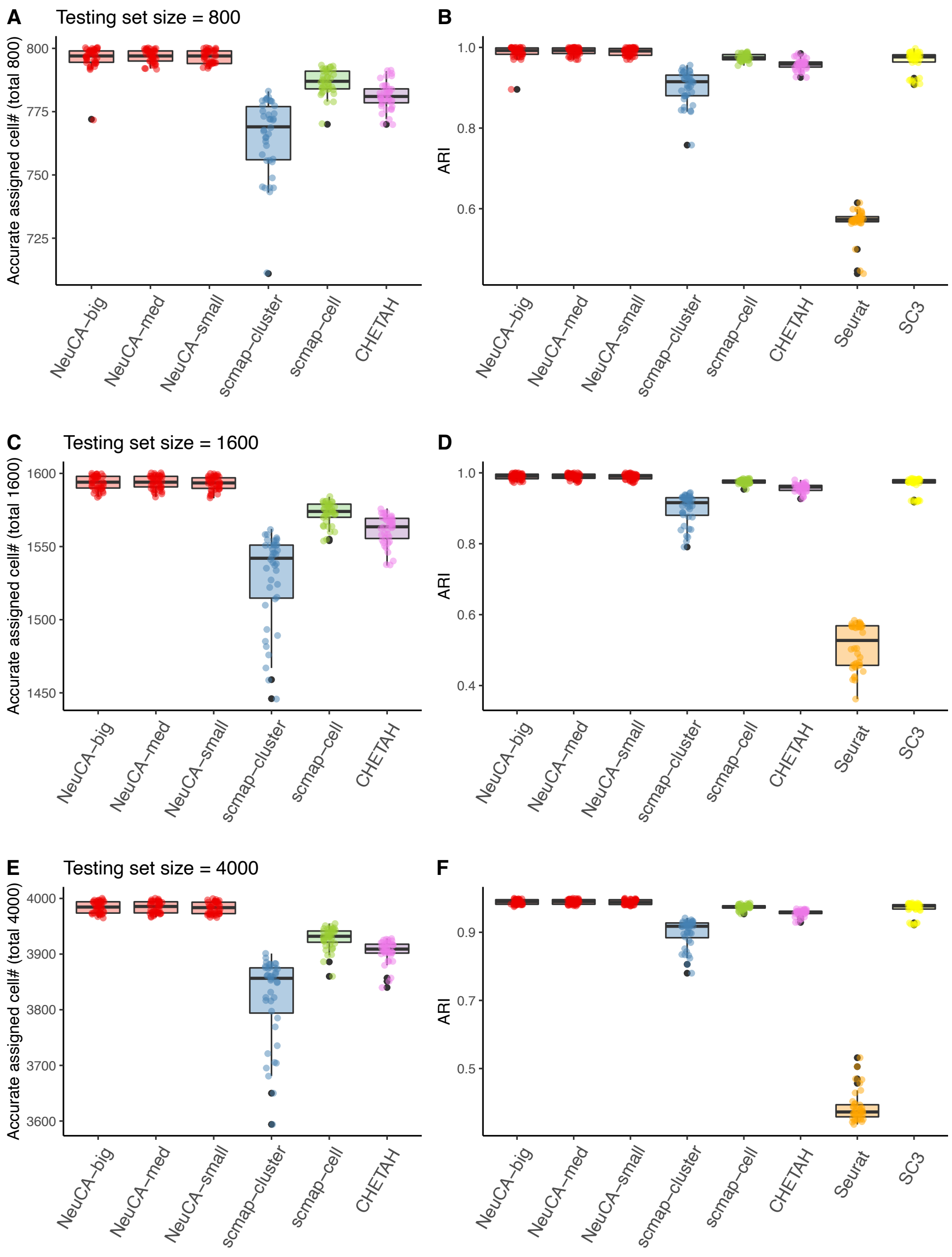

Supplementary Figure 5: Accurately assigned rate and ARI value using T-cell only data and different testing set size. From top to bottom, the testing set size increases from 800, 1600, to 4000. Results are summarized over different training size with each setting consisting of 20 Monte Carlo simulations.

Numerical experiments exclusively for "easy" PBMC dataset

Supplementary Figure 6 to 8 show NeuCA's performance for "easy" PBMC dataset. Here, all closely-correlated T cells were excluded from PBMC dataset, leading to very distinct cell types in the dataset that are easy to classify.

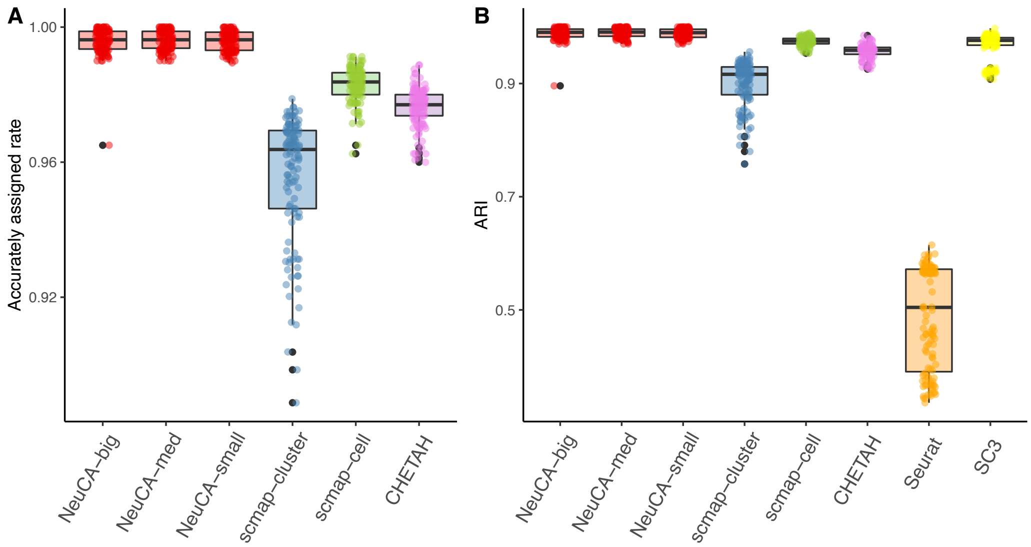

Supplementary Figure 6: Accurately assigned rate and ARI for applying the proposed method and existing methods on easy-PBMC dataset. Different training set sizes and testing set sizes are considered and summarized into one box for each method. 20 Monte Carlo simulations are used for each training/testing setting.

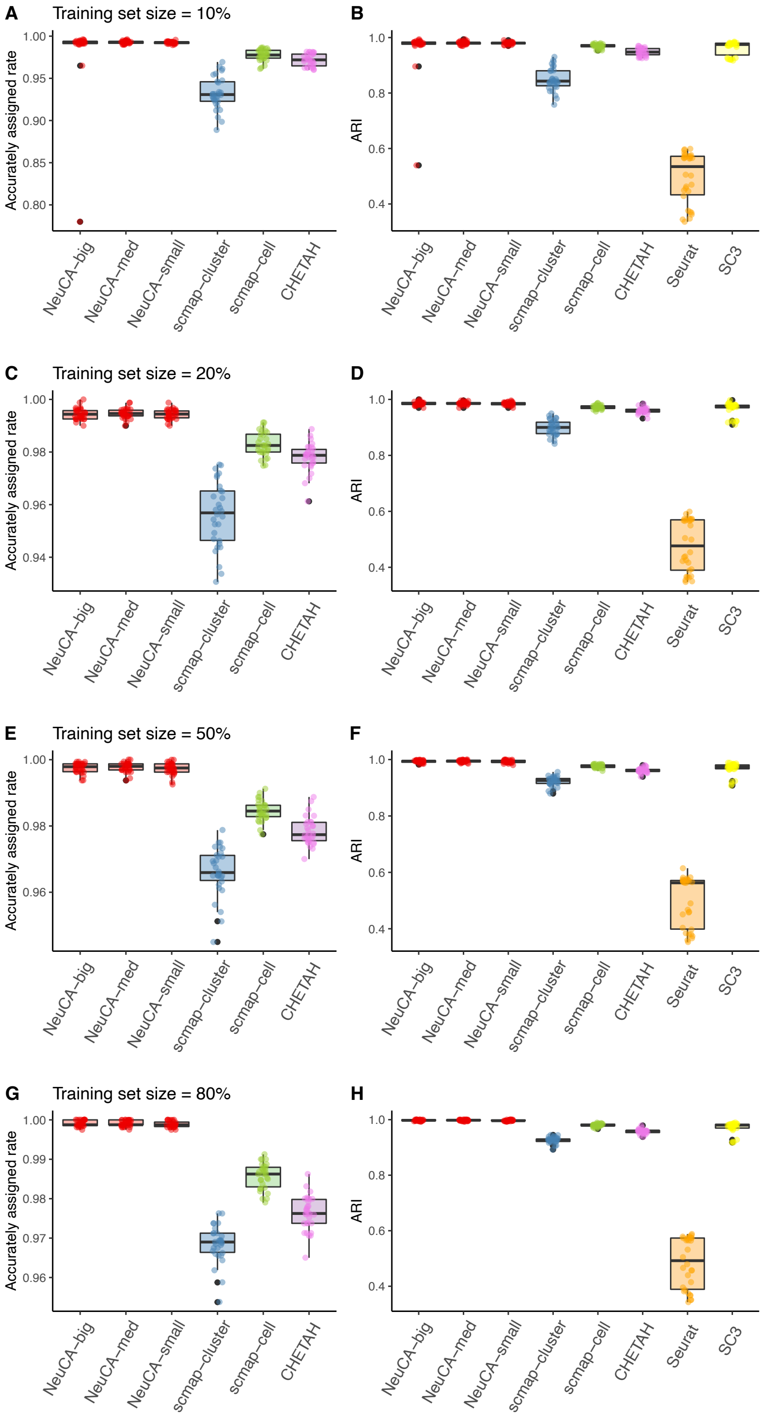

Supplementary Figure 7: Accurately assigned rate and ARI value using easy-PBMC data and different training set sizes. From top to bottom, the training set size increases from 10%, 20%, 50%, to 80%. Results are summarized over different testing size with each setting consisting of 20 Monte Carlo simulations.

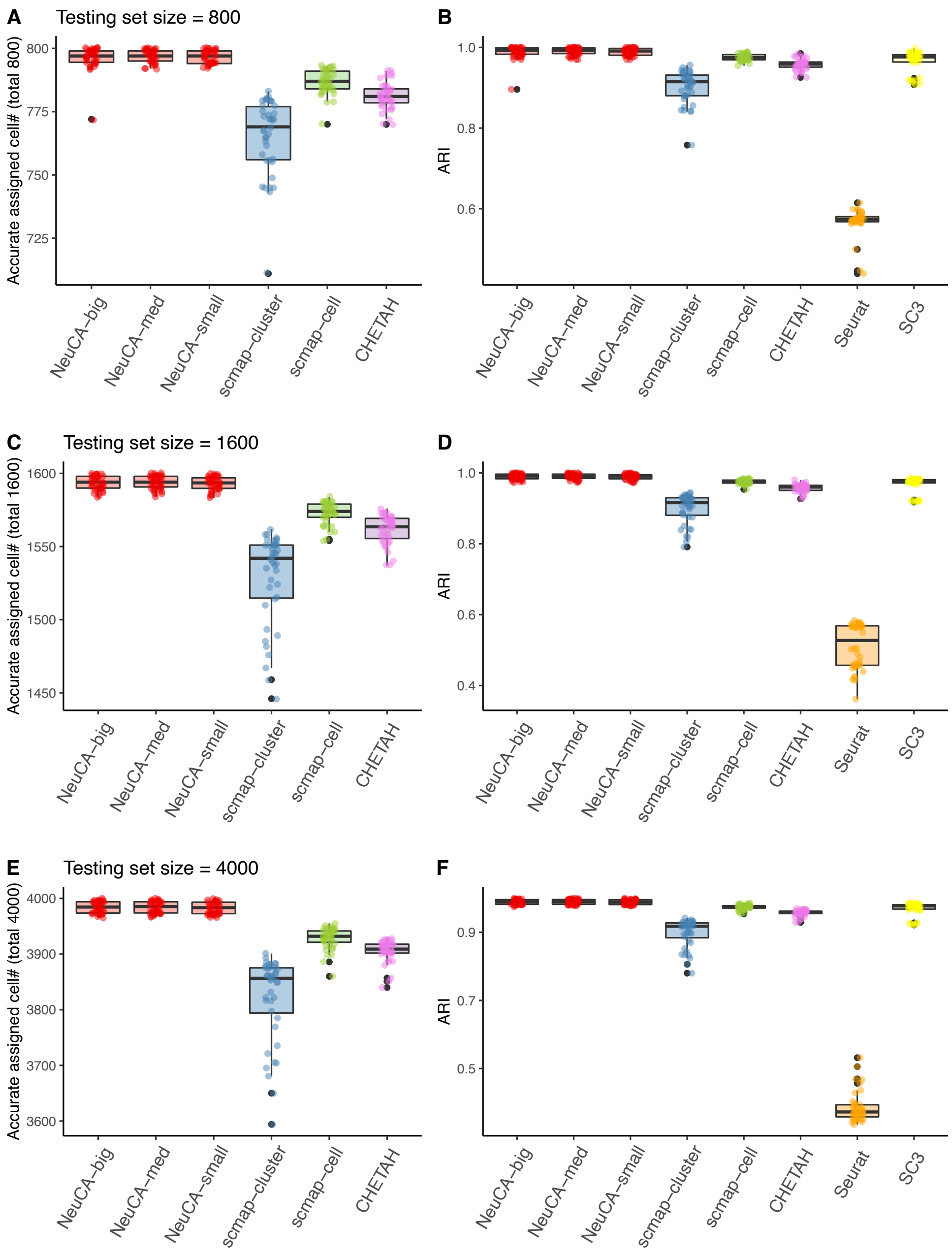

Supplementary Figure 8: Accurately assigned rate and ARI value using easy-PBMC data and different testing set size. From top to bottom, the testing set size increases from 800, 1600, to 4000. Results are summarized over different training size with each setting consisting of 20 Monte Carlo simulations.

Real data analysis with cell-sorted PBMC data

Supplementary Figure 9 and Supplementary Figure 10 are complementary to main Figure 3. They have misclassification rate represented as boxplot, for all supervised methods on PBMC dataset. They also have additional visualizations of cell type annotating and clustering results using scmap-cell, Seurat and SC3. It is the result on real cell-sorted PBMC data.

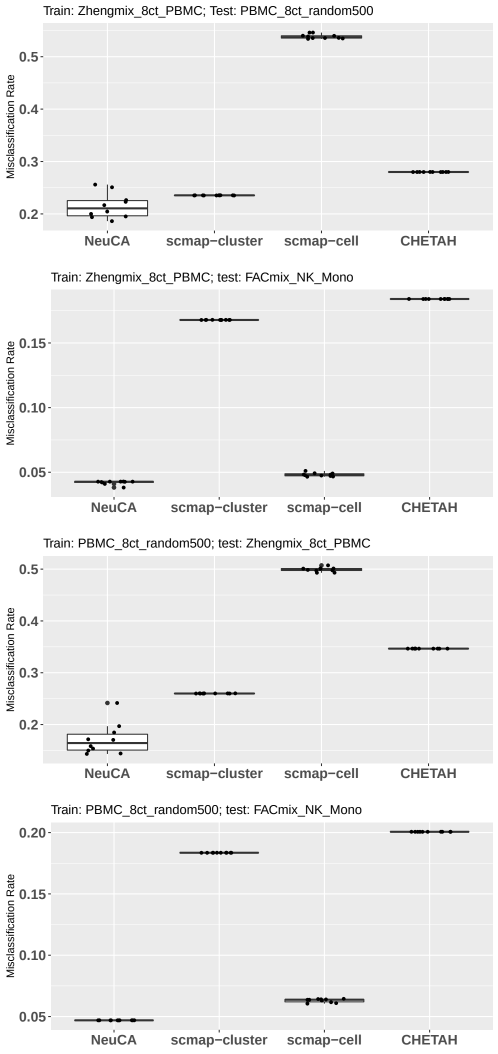

Supplementary Figure 9: Misclassification rates for all supervised methods on PBMC dataset. Rates are reported by alternating training and testing dataset used, in four settings.

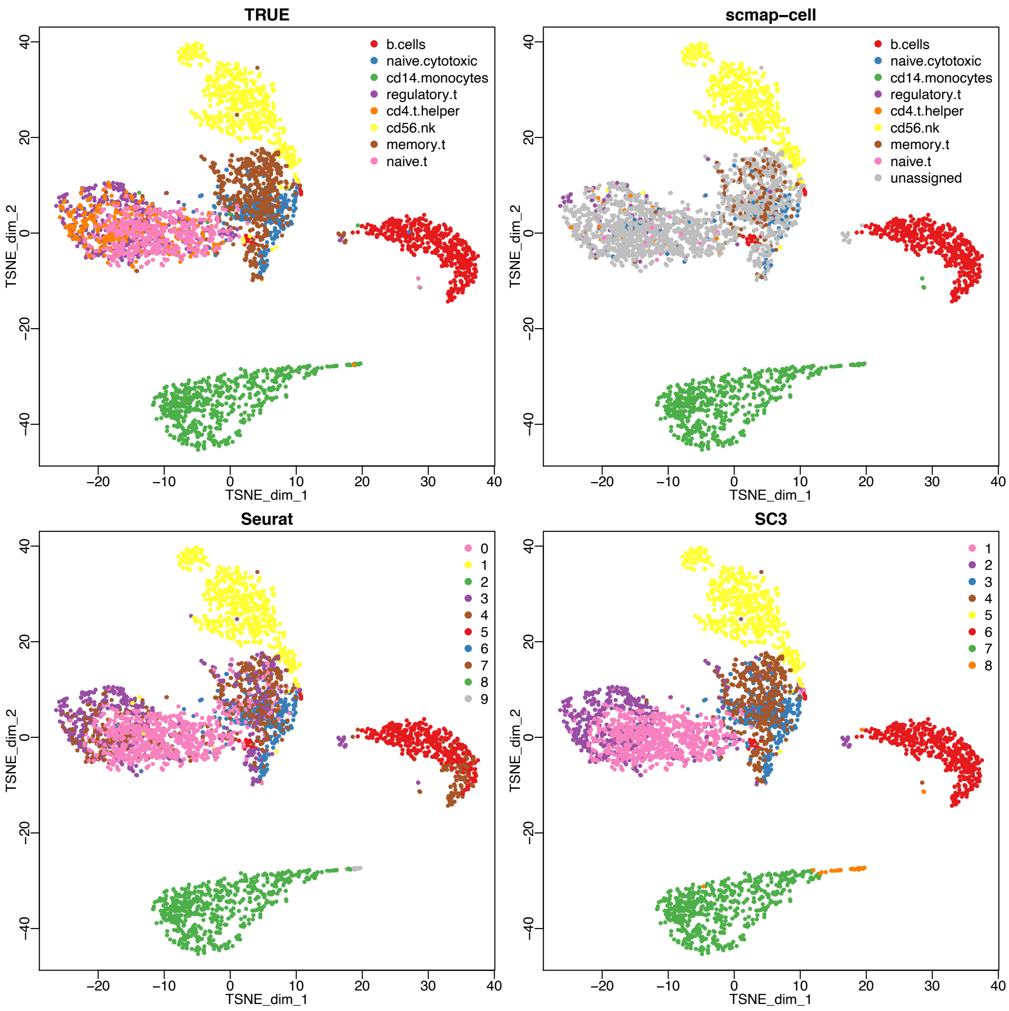

Supplementary Figure 10: Cell type annotating and clustering results from the PBMC real data experiment using scmap-cell, Seurat and SC3.

Real pancreas data analysis

Supplementary Figure 11, 12 and 13 are complementary to main Figure 4. They include misclassification rates for all combinations of training and testing dataset. And Sankey diagram of predicted cell labels of 4 different methods.

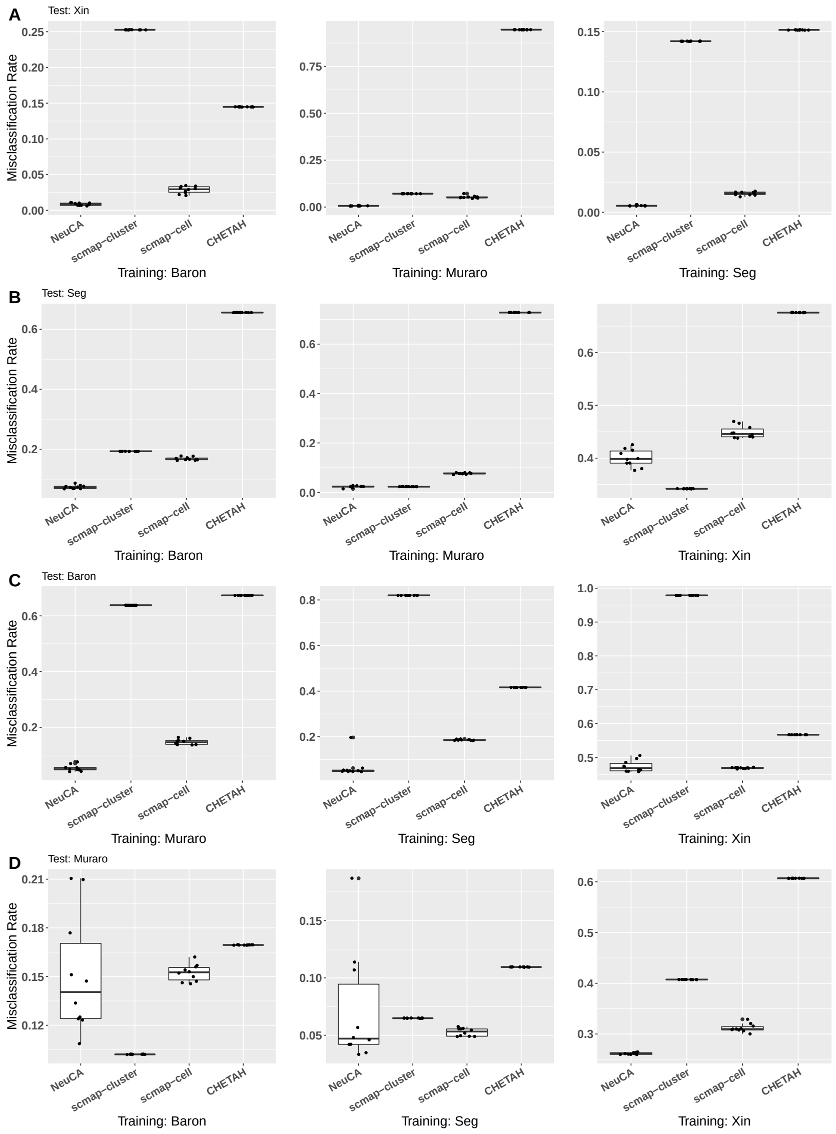

Supplementary Figure 11: Misclassification rates for all supervised methods on real pancrease dataset. Rates are reported by exhaustively listing all combinations of training and testing dataset.

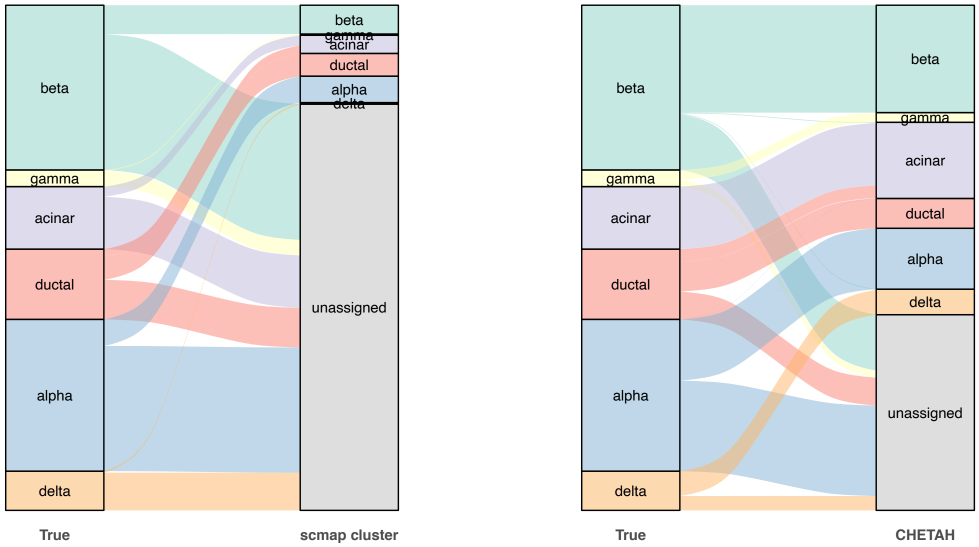

Supplementary Figure 12: Sankey plot of the true label and the estimated labels from pancreas data using scmap cluster and CHETAH. Seg data was used as training set and Baron data was used as testing set.

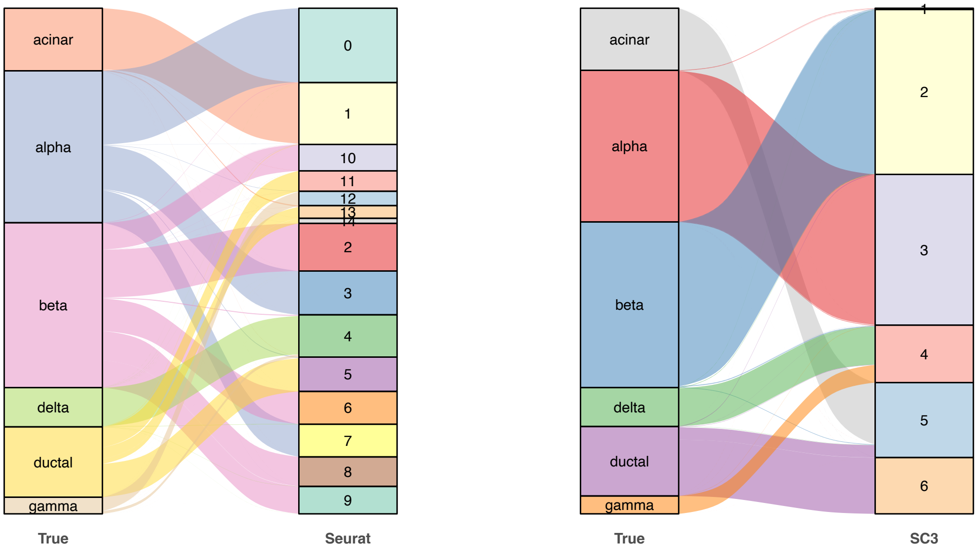

Supplementary Figure 13: Sankey plot of the true label and the estimated labels from pancreas data using Seurat and SC3. Seg data was used as training set and Baron data was used as testing set.

Real ASD data analysis

Supplementary Figure 14 is complementary to main Figure 5. They include misclassification rates for all combinations of training and testing dataset.

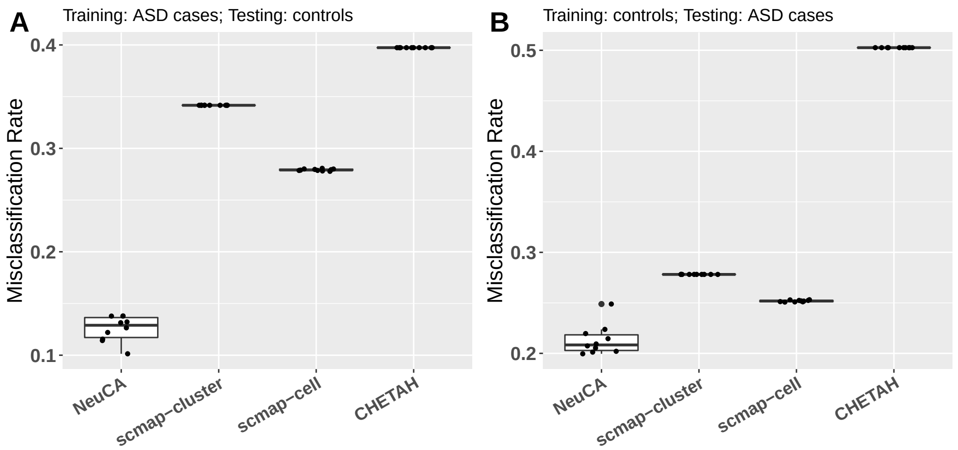

Supplementary Figure 14: Misclassification rates for all supervised methods on real ASD dataset. Results include both scenarios by alternating ASD cases data and controls data as training and testing.

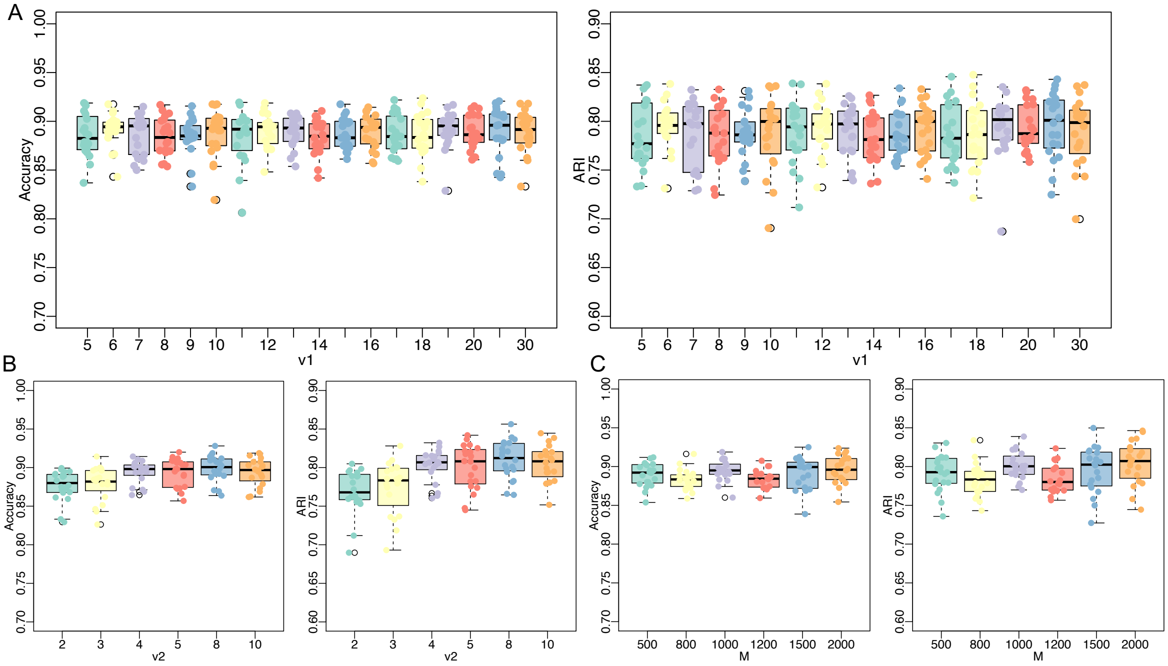

Supplementary Figure 15: Simulation results for using different tuning parameters. The simulation study is based on the setting using the cell sorted PBMC dataset with 8 cell types. Panel A fixed ν_2 and M at 3 and 1000 respectively, and changed ν_1 among $\{5, 6, 7, 8, 910, 11, 12, 13, 14, 15, 16, 17, 18, 19, 20, 25, 30\}$. Panel B fixed ν_1 at 10 and M at 1000, and changed ν_2 among $\{2, 3, 4, 5, 8, 10\}$. Panel C fixed ν_1 at 10 and ν_2 at 3, and changed M among $\{500, 800, 1000, 1200, 1500, 2000\}$. For all the panels, the left figure is the accuracy of the predicted labels using NeuCA versus the true labels, and the right figure is the adjusted rand index evaluation. For each tuning parameter combination, the results are summarized over 20 Monte Carlo simulations.

Benchmark with other neural-network based methods
We have compared NeuCA with alternative supervised neural-network methods for cell type prediction. The accuracy is shown in Supplementary Figure 16. We benchmarked NeuCA, a neural network method implementation, and scDeepSort [1]. NeuCA has comparable performance with scDeepSort, although leading by a small margin that is not significant. Also, NeuCA outperforms a neural network model with 4 hidden layers and 1,000 initial units. It is worth noting that NeuCA's run time is in several minutes, which is considerably faster than scDeepSort who took several hours each iteration (data not shown).

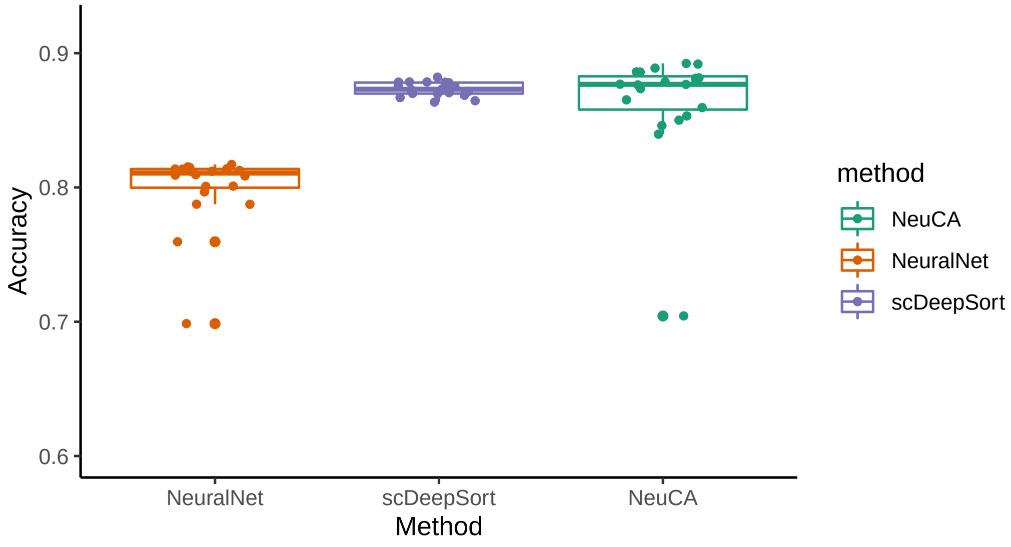

Supplementary Figure 16: Accuracy for three neural-network based methods for scRNA-seq cell type predition based on benchmark. Training and testing cells are randomly drawn from PBMC data used earlier. A total of 7 cell types are used in simulation. Each training dataset contains 5,000 cells per cell type. Each testing dataset contains 1,000 cells per cell type. Simulations are repeated for N=20 times.

References

[1] Xin Shao, Haihong Yang, Xiang Zhuang, Jie Liao, Penghui Yang, Junyun Cheng, Xiaoyan Lu, Huajun Chen, and Xiaohui Fan. scdeepsort: a pre-trained cell-type annotation method for single-cell transcriptomics using deep learning with a weighted graph neural network. *Nucleic Acids Research*, 2021.
